# Loss and Recovery of Glutaredoxin 5 Is Inducible by Diet in a Murine Model of Diabesity and Mediated by Free Fatty Acids In Vitro

**DOI:** 10.3390/antiox11040788

**Published:** 2022-04-15

**Authors:** Sebastian Friedrich Petry, Axel Römer, Divya Rawat, Lara Brunner, Nina Lerch, Mengmeng Zhou, Rekha Grewal, Fatemeh Sharifpanah, Heinrich Sauer, Gunter Peter Eckert, Thomas Linn

**Affiliations:** 1 Clinical Research Unit, Medical Clinic and Polyclinic III, Center of Internal Medicine, Justus Liebig University, 35392 Giessen, Germany; axel.roemer@ernaehrung.uni-giessen.de (A.R.); divyarawat21@gmail.com (D.R.); lara.s.brunner@med.uni-giessen.de (L.B.); nina.f.lerch@stud.mh-hannover.de (N.L.); mengmeng.zhou@med.uni-giessen.de (M.Z.); thomas.linn@innere.med.uni-giessen.de (T.L.); 2 Laboratory for Nutrition in Prevention & Therapy, Department of Nutritional Sciences, Justus Liebig University, 35392 Giessen, Germany; rekha.grewal@ernaehrung.uni-giessen.de (R.G.); gunter.eckert@ernaehrung.uni-giessen.de (G.P.E.); 3 Faculty of Medicine, Philipps University, 35037 Marburg, Germany; fatemeh.sharifpanah@gmail.com; 4 Cyntegrity Germany GmbH, 60438 Frankfurt, Germany; 5 Department of Physiology, Faculty of Medicine, Justus Liebig University, 35392 Giessen, Germany; heinrich.sauer@physiologie.med.uni-giessen.de

**Keywords:** glutaredoxin, β-cell, high fat diet, diabetes, obesity, mitochondria

## Abstract

Free fatty acids (FFA), hyperglycemia, and inflammatory cytokines are major mediators of β-cell toxicity in type 2 diabetes mellitus, impairing mitochondrial metabolism. Glutaredoxin 5 (Glrx5) is a mitochondrial protein involved in the assembly of iron–sulfur clusters required for complexes of the respiratory chain. We have provided evidence that islet cells are deprived of Glrx5, correlating with impaired insulin secretion during diabetes in genetically obese mice. In this study, we induced diabesity in C57BL/6J mice in vivo by feeding the mice a high-fat diet (HFD) and modelled the diabetic metabolism in MIN6 cells through exposure to FFA, glucose, or inflammatory cytokines in vitro. qRT-PCR, ELISA, immunohisto-/cytochemistry, bioluminescence, and respirometry were employed to study Glrx5, insulin secretion, and mitochondrial biomarkers. The HFD induced a depletion of islet Glrx5 concomitant with an obese phenotype, elevated FFA in serum and reactive oxygen species in islets, and impaired glucose tolerance. Exposure of MIN6 cells to FFA led to a loss of Glrx5 in vitro. The FFA-induced depletion of Glrx5 coincided with significantly altered mitochondrial biomarkers. In summary, we provide evidence that Glrx5 is regulated by FFA in type 2 diabetes mellitus and is linked to mitochondrial dysfunction and blunted insulin secretion.

## 1. Introduction

Visceral obesity is one of the major risk factors for developing glucose intolerance and type 2 diabetes mellitus. A sedentary lifestyle combined with Western dietary habits promotes diabetes in genetically predisposed individuals. A misbalance of calorie intake and muscular activity leads to sustained nutritional overflow in tissues with predominantly insulin-dependent glucose uptake. An insulin-resistant metabolism is hallmarked by hyperglycemia, elevated free fatty acids (FFA), and a chronic state of inflammation mediated through cytokines such as interleukin-1β (IL-1β), interferon-γ (IFN-γ), and tumor necrosis factor-α (TNF-α) [1]. This adverse metabolic condition challenges the pancreatic β-cells which are imperative for maintaining glucose homeostasis. They feature a marked metabolic activity and therefore rely on a vast amount of energy supplied by their mitochondria, especially when a supraphysiologically high level of insulin secretion is required to overcome insulin resistance [2] and ameliorate hyperglycemia. This immense substrate turnover in the respiratory chain promotes the formation of superoxide and hydrogen peroxide at complexes I–III [3], eventually leading to the functional and structural decay of mitochondria [4]. Ultimately, the secretory apparatus of the β-cells is impaired. The excess of FFA further fuels these processes by promoting the formation of reactive oxygen species (ROS) through peroxisomal β-oxidation [5], the phosphorylation of insulin receptor substrates [6], the induction of the p53-dependent mitochondrial apoptotic pathway [7], and interference with the cell cycle [7]. Activated FFA form acyl-CoA, which accumulates due to mitochondrial dysfunction, activating PPARγ and the sterol regulatory element-binding protein (SREBP)-1c-dependent pathways with detrimental effects on insulin release, cell proliferation and survival, and leads to an increased uncoupling of mitochondria [8].

Glutaredoxin 5 (Glrx5) is a mitochondrial enzyme of the thioredoxin family. It is an acknowledged actor in the iron–sulfur (Fe/S) cluster assembly and consecutively for the biogenesis of enzymes containing these clusters [9,10]. Glrx5, with a Cys-Gly-Phe-Ser active site, binds Fe/S clusters and transfers them to specific target apoproteins where they are required for structural conformation, electron transfer, or as co-substrates (for an overview, see [11]). Fe/S clusters form reactive centers in complexes I–III of the respiratory chain and prosthetic groups in cytosolic aconitase (Aco1) and mitochondrial ferrochelatase. In several cell models including human cells, it has been demonstrated that Glrx5 deficiency leads to an increased susceptibility to oxidative and osmotic stress and cellular iron overload [12,13,14,15]. A human subject suffering from Glrx5 deficiency was reported to present anemia, type 2 diabetes mellitus/type 3c diabetes mellitus (i.e., by damage to the exocrine pancreas), hepatosplenomegaly, and cirrhosis of the liver due to cellular iron overload [16]. The application of iron chelators markedly ameliorated these conditions in line with data derived from a murine model of cellular iron overload [17]. Free cellular iron induces oxidative damage to membrane lipids and the formation of lipid peroxides, resulting in oxidative, non-apoptotic cell decay known as ferroptosis [18].

Little is known about the role of Glrx5 in pancreatic β-cells and its regulation. Therefore, we employed a dietary-induced model of murine diabesity to study the expression of Glrx5 in pancreatic islets under a high-fat diet (HFD) versus standard chow. The animals featured a considerable loss of islet Glrx5 concomitant with elevated serum FFA, obesity, and impaired glucose tolerance after HFD feeding. In vitro, murine β-cells were exposed to fatty acids, high glucose, or inflammatory cytokines. The nutritional regimen employed in vivo was modelled by pre-incubation with free fatty acids followed by exposure to high glucose. Only culture conditions involving FFA led to a decline in Glrx5 and impaired mitochondrial metabolism.

In summary, this study delivers in vivo and in vitro evidence for a distinct regulation of islet Glrx5 by FFA and links the loss of Glrx5 with failing insulin secretion due to impaired mitochondrial metabolism.

## 2. Materials and Methods

### 2.1. Research Animals

A total of 88 male C57BL/6J mice were acquired from Charles River (Sulzfeld, Germany) at the age of 5 weeks and were given two weeks to adapt to the local animal facility. They were housed at 22 ± 2 °C, with a 14:10 h light/dark cycle, a relative humidity of 55 ± 10%, and provided with tap water ad libitum in individually ventilated cages in groups of five mice. All animal research was carried out in accordance with recommendations from our institutional animal welfare officer, the Chair of Animal Welfare of the Justus Liebig University, Giessen, and the Regional Administrative Council of Giessen, the Veterinary Department, under codes GI 20/11 No. G 3/2017 and No. G 48/2018. Both committees approved the protocol. All experiments were performed in accordance with the German Animal Welfare Law.

**Figure 1:**
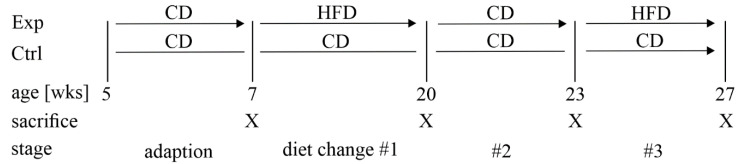
Experimental setup of the animal experiment. Male C57BL/6J mice were divided into the experimental (Exp) and control (Ctrl) groups at the age of 7 weeks after 2 weeks of adaption to our animal housing. Control animals were fed a control diet (CD, ca. 3514 kcal/kg, 10% of energy from fat, 66% from carbohydrates, and 24% from protein) throughout the study period. The experimental group was fed a high-fat diet (HFD, ca. 5389 kcal/kg, 70% of energy from fat, 14% from carbohydrates, and 16% from protein) from 7 to 20 weeks of age, followed by rescue feeding with the CD for 3 weeks, and another cycle of the HFD for 4 weeks. Euthanasia was carried out in both groups at 7, 20, 23, and 27 weeks of age.

During adaption to our animal facility, both groups of mice were fed a control diet (CD, 3514 kcal/kg, 10% of energy from fat, 66% from carbohydrates, and 24% from protein; diet C 1090-10, Altromin, Lage, Germany) ad libitum. The control group did not undergo a change of diet throughout the study period of 22 weeks. The experimental group received a high-fat diet (HFD, 5389 kcal/kg, 70% of energy from fat, 14% from carbohydrates, and 16% from protein; diet C 1090-70, Altromin, Lage, Germany) for 13 weeks (from 7 to 20 weeks of age). Afterwards, the experimental group was switched back to the CD until 23 weeks of age (3 weeks in total) before it was fed the HFD again for 4 more weeks (age 23 to 27 weeks). A summary of the experimental setup is given in Figure 1.

Blood glucose levels were monitored with a glucometer (OneTouch Ultra 2, LifeScan, Düsseldorf, Germany) using blood samples collected by puncturing the tip of the tail after overnight fasting. Animals of both groups were euthanized at 7, 20, 23, and 27 weeks of age. Intraperitoneal glucose tolerance tests (IPGTT) were conducted at these time points. The fasting blood glucose level was measured before glucose (1 mg per g body weight) was injected intraperitoneally and 30, 60, 90, and 120 min thereafter.

### 2.2. Pancreatic Islet Isolation

Mice were anesthetized with 1 mg and 0.2 mg of ketamine (Medistar, Ascheberg, Germany) and xylazine (Ceva, Düsseldorf, Germany), respectively, which were dissolved in 0.9% NaCl per 10 g of body weight and administered via intraperitoneal injection. To harvest the pancreas in ischemia, the abdominal wall was excised, the aorta was cut, and the blood was drained and collected for further analysis before the removal and processing of the pancreas for either immunohistology or islet isolation.

For immunohistochemistry, organs were fixed with 4% phosphate-buffered paraformaldehyde in picric acid (pH 7.3) following the protocol established by Stefanini et al. [19] for four hours, then washed and stored in PBS supplemented with 18% sucrose solution overnight. Organs were embedded in a cryoblock embedding medium (Biosystems, Nunningen, Switzerland) and frozen at −80 °C.

Islet isolation was carried out as described previously by our department [20]. Briefly, the pancreas was perfused with 4 mg/mL collagenase B (Roche, Mannheim, Germany) dissolved in 1% Hank’s solution (Biochrom, Berlin, Germany) and supplemented with 35 mL HEPES buffer (Biochrom), 10 mL ciprofloxacin, 10 mL penicillin–streptomycin, and 1 mL gentamycin through the ductus pancreaticus. To facilitate the process of digestion, the perfused organ was mechanically chopped with a pair of scissors before incubation in a collagenase solution at 37 °C in a shaking water bath for 10 min. Every 3 min, tissue was vortexed for 10 s. Digested tissue was then shaken by hand for another 2 min, and the process was eventually stopped by adding cold Hank’s solution. Following 3 min of centrifugation at 375 g, the supernatant was discarded, and the pellet was dissolved in 15% Parker/fetal calf serum dissolved in Medium 199 (Gibco, Darmstadt, Germany) and fetal bovine serum (Biowest, Nuaillé, France) at room temperature. Islets were hand-picked under a stereomicroscope and incubated overnight at 37 °C to overcome isolation stress.

### 2.3. Immunohistochemistry

Immunohistological staining was employed for the detection of insulin and Glrx5. Frozen organs were thawed and sectioned entirely. Slides with a thickness of 7 µm were created using a Leica Cryostat CM1850 (Leica, Wetzlar, Germany). A manual optical assessment for quality was employed and slides with a damaged structure were rejected. An interval of 140 µm between slides was maintained to avoid the multiple inclusion of islets. Slides were washed with PBS and blocked with 1% donkey serum dissolved in PBS containing 0.3% Triton X-100 (0.3% PBST) for 20 min. Sections were incubated with respective primary antibodies (insulin: 1:100, Bio-Rad, Feldkirchen, Germany; Glrx5: 1:100, Bioss, Woburn, MA, USA) diluted in 1% donkey serum dissolved in 0.3% PBST overnight at 4 °C in a wet chamber. Secondary antibodies (1:400, FITC and Rhodamine Red, Jackson, Newmarket, UK) in 5% mouse serum were applied for one hour at room temperature in the dark before nuclei were stained with Hoechst 33,342 (Calbiochem, Darmstadt, Germany) in 0.1% TRIS buffer with a pH of 7.6, and samples were preserved with ProLong Gold (Invitrogen, Darmstadt, Germany). Slides without primary antibodies were used as negative controls.

Images of stained samples were taken with Leica Application Suite V 3.8.0 using a DFC420 C digital microscope camera (Leica, Wetzlar, Germany). The ImageJ software (Wayne Rasband, National Institutes of Health, Bethesda, MD, USA) was used for this analysis. The islet area and the respective staining area of the insulin or Glrx5 were measured by the optical identification of islets and manual selection by use of the freehand selection tool after calibrating ImageJ to match the scale. The absolute staining area of the insulin or Glrx5 staining inside the respective islets was then acquired by applying a threshold value and manual adaption to exclude regions without staining. The staining of Glrx5 was quantified by dividing its staining area by the area of insulin staining for each islet.

### 2.4. ROS Production Analysis

Intracellular ROS production in islets was detected employing the 2′,7′-dichlorofluorescein diacetate (DCFH-DA, Sigma-Aldrich, Taufkirchen, Germany) staining method as previously described [21,22,23]. Briefly, isolated primary islets were incubated in the dark in a serum-free medium containing a 10-µM DCFH-DA indicator dye dissolved in dimethylsulfoxide (DMSO, Sigma-Aldrich) for 30 min at 37 °C. Samples were rinsed with a pre-warmed serum-free medium and immediately analyzed. DCF fluorescence was evaluated in 3600 μm^2^ regions of interest by a confocal laser scanning microscope (Leica SP2 AOBS, Bensheim, Germany) and the 488 nm band of its argon ion laser. Emission was measured using a long-pass LP515 nm filter set. The fluorescence was quantified with the Leica Simulator software using an overlay mask and correction for background fluorescence.

### 2.5. mRNA Expression Analysis

RNA was extracted from isolated islets using the RNeasy Plus Micro Kit (Qiagen, Düsseldorf, Germany). A NanoDrop 1000 spectrophotometer (Thermo Scientific, Schwerte, Germany) was employed to determine the total RNA concentration (OD 260/280 nm). cDNA was synthesized with the SuperScript III Reverse Transcriptase kit (Invitrogen). Quantitative real-time PCR (qRT-PCR) was carried out on the StepOnePlus Real-Time PCR System (Applied Biosystems, Waltham, MA, USA) conducting activation for 10 min at 95 °C, 40 cycles of denaturation (95 °C for 10 s), and carrying out annealing/extension at 60 °C for 1 min. The primer concentration was 10 µM. The sequences of the employed primers (Invitrogen) were as follows:

Beta-actin (reference):

fwd GTG GGA ATG GGT CAG AAG G,

rev GAG GCA TAC AGG GAC AGC A;

Ins1:

fwd TAT AAA GCT GGT GGG CAT CC,

rev GGG ACC ACA AAG ATG CTG TT;

Glrx5:

fwd GAA GAA GGA CAA GGT GGT GGT CTT C,

rev GCA TCT GCA GAA GAA TGT CAC AGC

Relative mRNA expression values were obtained by normalizing the respective Ct values of target genes with the reference gene using the delta Ct method. Delta delta Ct values were calculated for the controls vs. the HFD group for each time point and the fold change in mRNA expression was calculated.

### 2.6. Glucose Stimulation of Pancreatic Islets

Islets were isolated from the HFD and control mice as described above, and were hand-picked, transferred to a petri dish with TCM-199 (Biochrom) with a low glucose medium (30 mg/dL), and incubated for 30 min at 37 °C. They were then further incubated with 30 mg/dL or 300 mg/dL glucose in TCM-99 at 37 °C in a shaking water bath for 90 min. A total of five islets of equivalent size were used per condition. After incubation, the supernatant was collected and islets were sonicated in an immunoreactive insulin (IRI) buffer (7.12 g Na_2_HPO_4_ × H_2_O, 6 g NaCl, 3 g BSA, in 1000 mL distilled water) for lysis. Samples were stored at −20 °C before analysis.

### 2.7. Cell Culture

The employed murine β-cell line, mouse insulinoma 6 (MIN6), was maintained in Dulbecco’s Modified Eagle Medium (DMEM, Gibco) enriched with 25 mM glucose, 1 mM pyruvate, 15.8% heat-inactivated fetal calf serum (Biowest, Nuaillé, France), 50 µM 2-mercaptoethanol (Life Technologies, Carlsbad, CA, USA), and 80 U/mL penicillin/streptomycin (Life Technologies), as previously described by our group [24,25]. We used cells at passages 50–60 grown up to a confluence of 70–80%. The culture conditions were 37 °C and 5% CO_2_. The culture media were replaced every 2–3 days. Splitting was carried out by trypsinization with 0.05% trypsin–EDTA (Gibco) after washing with PBS. Beforehand, seeding cells were detached by applying DMEM and centrifugation at 250 *g* for 4 min.

Oleic (Enzo Life Sciences, Lörrach, Germany) and palmitic acid (Sigma-Aldrich) were dissolved in absolute ethanol (Sigma-Aldrich, with a purity higher than 99.8%) to prepare stock solutions of 300 mM and were added to DMEM (Gibco) with fatty acid-free BSA fraction V (Sigma-Aldrich) to reach final fatty acid concentrations of 0.75, 1.5, and 3 mM. The molar FFA:BSA ratio was 4:1. After being overlayed with nitrogen (Linde, Pullach, Germany) overnight at 37 °C on a shaker and sterile filtration, the stock solutions were stored at −20 °C. The ethanol concentrations for 0.75, 1.5, and 3 mM of final fatty acid solution were 0.23, 0.45, and 0.9%, respectively. The control medium was prepared to match the respective alcohol and BSA concentrations. For treatment with cytokines, 10 ng/mL TNF-α, 5 ng/mL IL-1β, and 100 ng/mL IFN-γ (all from R&D Systems) were dissolved in a standard cell culture medium. For the glucose treatment, the culture medium was replaced by a fresh medium without glucose for 2 h, after which it was renewed and enriched with the respective concentrations of glucose. MIN6 cells were pre-incubated with palmitic acid (0 or 400 µM for 48 h) prior to starving them in a Krebs–Ringer–HEPES (KRH) buffer (129 mM NaCl, 5 mM NaHCO_3_, 4.8 mM KCL, 2.5 mM CaCl_2_, 1.2 mM MgSO_4_ and 0.1% (*w*/*v*) BSA, 10 mM HEPES, pH 7.4) for 1 h followed by exposing the cells to glucose (0, 3, or 16 mM) for 1 h.

### 2.8. Cell Metabolic Activity

The cell metabolic activity was assessed by a 3-(4,5-dimethylthiazol-2-yl)-2,5-diphenyltetrazolium bromide (MTT, Abcam, Cambridge, UK, purity > 98%) assay. We employed 10^4^ MIN6 cells in 200 µL DMEM attached to a 96-well plate. The medium was replaced with a fatty acid medium for 24 h. After treatment, 50 µL of the medium was replaced by 2 mg/mL MTT freshly dissolved in PBS to achieve a final concentration of 0.5 mg/mL, and then sterile filtered (0.22 μm) and incubated at 37 °C in 5% CO_2_. After 4 h, the medium was replaced by 200 µL DMSO. After formazan crystals were dissolved for 15 min on a plate shaker, the absorbance was measured at 570 nm and 620 nm (background) using a multimode microplate reader (Berthold Technologies, Bad Wildbad, Germany).

### 2.9. Immunocytochemistry

For immunocytochemistry, adherent cells were trypsinized using 0.05% trypsin-EDTA. Cells were collected and centrifuged at 250 *g* for 4 min before seeding them on petri dishes where treatment was conducted for 24 h. The medium was removed, and the cells were washed with PBS and trypsinized in an incubator at 37 °C for 2–3 min. Detached cells were centrifuged in PBS at 250 *g* for 4 min. The cell pellets were diluted with PBS, and 10–20 µL containing 2000–5000 cells were dropped on slides. The cells were dried for 20–40 min and stored at −20 °C before staining was conducted, following the protocol described for immunohistology. The protein levels of insulin and Glrx5 were quantified with ImageJ using the mean fluorescent intensity normalized by the cell area. This resulted in the integrated density (IntDen), as previously described [26]. Briefly, all eligible single cells were selected manually. The IntDen, area, and mean fluorescent intensity of the background were measured. The IntDen was normalized by subtracting the product of the cell area and mean background fluorescent intensity.

### 2.10. Determination of Cellular ATP Levels

Twenty-four hours prior to incubation, 5 × 10^4^ cells/well were seeded into 96-well luminescence plates (Corning Inc., Corning, NY, USA). The cells were incubated with oleic acid (0.75, 1.5, or 3 mM) or ethanol as the solvent control for 24 h. ATP levels were determined using a luciferase-based bioluminescence assay. Cells were lysed by a NaOH lysis buffer, followed by adding a solution containing luciferase, luciferin, and co-substrates (PerkinElmer, Waltham, MA, USA). The luminescence was measured using a multimode microplate reader (CLARIOstar, BMG LABTECH, Ortenberg, Germany).

### 2.11. Mitochondrial Respiration

Respiratory states were measured at 37 °C using the Oxygraph-2k (OROBOROS Instruments, Innsbruck, Austria) based on a silver chloride (Clarke) electrode and DatLab software version 7.0.0.2 (OROBOROS Instruments). For the analysis of the respiratory system, a complex multiple substrate–uncoupler–inhibitor titration (SUIT) protocol (elaborated by Prof. Dr. Erich Gnaiger, University of Innsbruck [27]) was applied including different substrates, uncouplers, and inhibitors. After 24 h of treatment with 0.75 mM oleic acid or respective ethanol solvent controls, cells were harvested in mitochondrial respiration medium MIR05 (0.5 mM EGTA, 3 mM MgCl_2_, 60 mM K-lactobionate, 20 mM taurine, 10 mM KH_2_PO_4_, 20 mM HEPES, 110 mM sucrose, 1 mg/mL BSA, pH 7.1) developed by OROBOROS [28]. The cells were centrifuged and resuspended in MIR05 to obtain a solution containing 5 × 10^5^ cells/mL. After adding 2 mL of cell suspension into each chamber of the oxygraph, the respiration stabilized (endogenous respiration). The cell membranes were permeabilized with digitonin (5 µL/10^6^ cells), leaving the mitochondrial outer and inner membranes intact. The capacity of the oxidative phosphorylation (OXPHOS) of complexes I and II was determined by adding complex I substrates glutamate (10 mM) and malate (2 mM), and complex V substrate ADP (2 mM) followed by the complex II substrate succinate (10 mM). Adding the complex V inhibitor oligomycin (2 µg/mL) led to a leak in the respiration. Uncoupled electron transfer system (ETS) capacity was achieved by the titration of protonophore FCCP. After adding the complex I inhibitor rotenone (0.5 µM), uncoupled complex II respiration was achieved. The inhibition of complex III by applying antimycin A (2.5 µM) determined the residual oxygen consumption (caused by enzymes which do not belong to the electron transfer system), which was subtracted from all respiratory states. By adding N,N,N′,N′-tetramethyl-p-phenylenediamine (0.5 mM) and ascorbate (2 mM), complex IV was measured. The autoxidation rate was determined using sodium azide (≥100 mM). Complex IV respiration was additionally corrected for autoxidation [29]. The results of mitochondrial respiration were normalized with the activity of citrate synthase measured by the conversion of DTNP into TNP at 412 nm by a GENESYS 10S photometer (Thermo Fisher Scientific, Waltham, MA, USA).

### 2.12. ELISA Analysis

ELISA was employed for the quantitative analysis of protein content in cell and islet lysates and medium and FFA levels in murine blood. Commercial kits were used according to the respective manufacturers’ instructions: insulin (DRG Diagnostics, Marburg, Germany), Glrx5 (CUSABIO Technology, Houston, TX, USA), and FFA (Abcam, Cambridge, UK). Prior to the cell lysis of 4 × 10^5^ MIN6 cells per condition, 1 mL of culture medium was extracted, centrifuged at 330 g for 5 min at 4 °C, and supernatant was preserved for analysis. Cells were washed twice in ice-cold PBS and incubated on ice for 20 min in 10% NP-40 alternative lysis buffer (Merck Millipore, Burlington, MA, USA) with 3% *v/v* proteinase inhibitor 100× (Halt, Thermo Fisher Scientific, Waltham, MA, USA). Cell debris was removed by centrifugation at 12,000 *g* at 4 °C for 15 min, leaving the supernatant for further use. Pancreatic islets were prepared as described above. The amount of insulin and Glrx5 protein was normalized to the total lysate protein as determined by the Bradford protein assay (Bio-Rad Laboratories, Hercules, CA, USA).

### 2.13. Statistical Analysis

The statistical analysis was performed using GraphPad Prism 7 (GraphPad Software, San Diego, CA, USA) using an unpaired *t*-test or one- or two-way ANOVA with Dunnett or Sidak multiple comparison tests when appropriate. The Pearson correlation coefficient was calculated for correlation analyses. Data are given as mean values ± SEM, unless otherwise stated. A *p*-value of <0.05 was considered significant.

## 3. Results

### 3.1. A Mouse Model of Dietary-Induced Diabesity

**Figure 2:**
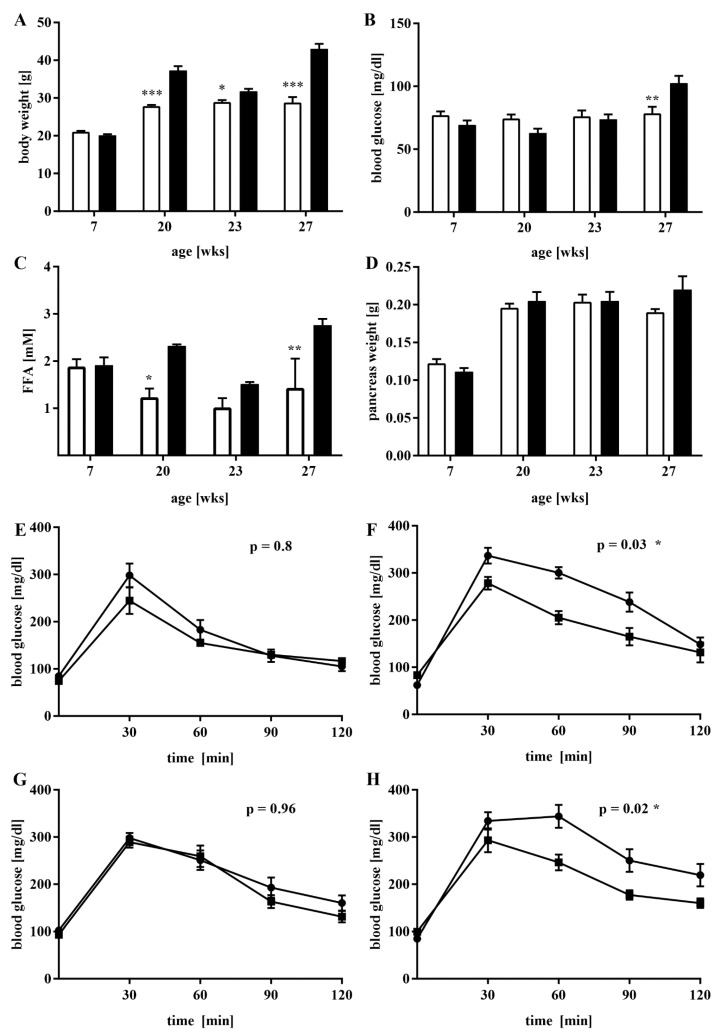
Fasting and dynamic blood glucose levels, body weight, free fatty acid levels, and pancreas weight of the research animals. IPGTT and euthanasia was conducted before dietary changes and at the end of the experimental procedure at 7, 20, 23, and 27 weeks of age. Fasting blood glucose levels and body weight were monitored every two weeks until 20 weeks of age, and then weekly. (**A**) Body weight and (**B**) fasting blood glucose values at 7, 20, 23, and 27 weeks of age; *n* = 5–33 mice/timepoint/group. (**C**) Blood FFA levels as determined by ELISA; *n* = three mice/timepoint/group. (**D**) Pancreas weight; *n* = six mice/timepoint/group. (**E**–**H**) IPGTT results at (**E**) 7, (**F**) 20, (**G**) 23, and (**H**) 27 weeks of age; *n* = five mice/timepoint/group. For each graph, the white bars represent the controls, and the black bars represent the experimental group. *** denotes *p* < 0.001, ** *p* < 0.005, and * *p* < 0.05 ((**A**–**D**): two-way ANOVA, (**E**–**H**): unpaired *t*-test).

To study the effect of the dietary-induced abundance of FFA on the expression of Glrx5 in the islets of Langerhans in C57BL/6J-mice, the mice were switched from a high-fat diet to standard chow and back during the study period (as described in Materials and Methods and Figure 1). The control mice gained weight physiologically as they aged, whereas the animals from the experimental group gained weight quickly when fed the HFD. During the switch to the CD from 20 to 23 weeks of age, a rapid weight loss was observed before another increase in body weight after the second phase of the HFD. After both stages with the HFD at 20 and 27 weeks, the mice exhibited a significantly higher body weight compared to the control group (27.85 ± 1.59 vs. 37.3 ± 5.89 g, *** *p* < 0.001 at 20 weeks; 28.8 ± 3.19 vs. 43 ± 3 g, *** *p* < 0.001 at 27 weeks, Figure 2A). Along with this, the amount of FFA in the blood of the experimental animals increased from 20 weeks of age onwards, especially after both stages of HFD feeding at 20 and 27 weeks of age (1.24 ± 0.32 vs. 2.32 ± 0.06 mM, * *p* < 0.05 at 20 weeks; 1.43 ± 1.07 vs. 2.77 ± 0.23 mM, ** *p* < 0.005 at 27 weeks, Figure 2C). The fasting blood glucose levels were only significantly elevated at the end of the study at 27 weeks of age (78.56 ± 20.53 vs. 102.6 ± 22.76 mg/dL, ** *p* < 0.005, Figure 2B). The dynamic glucose metabolism, as measured by IPGTT, showed obvious signs of impairment after HFD feeding. Although no difference between respective blood glucose values was detected when both groups of mice were compared at the beginning of the study at seven weeks of age (AUC 18,732 ± 1518 vs. 21,107 ± 1880 mg/dL x min, n.s., Figure 2E), HFD-fed mice showed significantly higher glucose levels at 20 weeks of age (AUC 22,667 ± 1610 vs. 29,403 ± 1512 mg/dL x min, * *p* < 0.05, Figure 2F) as a sign of an impaired first phase of glucose-stimulated insulin secretion (GSIS). When switched to the CD, this difference could no longer be detected (AUC 24,703 ± 1604 vs. 26,180 ± 1761 mg/dL x min, n.s., Figure 2G). After four more weeks of HFD feeding, the respective mice exhibited significantly worse glycemic control (AUC 25,377 ± 1732 vs. 32,393 ± 2211 mg/dL x min, * *p* < 0.05, Figure 2H). Neither pancreas weight (Figure 2D) nor islet count (data not shown) differed significantly between both groups, although a trend in higher organ weight and increased islet count was detected towards the end of the study.

### 3.2. The Islet Glrx5 Content Is Diet-Dependent

**Figure 3:**
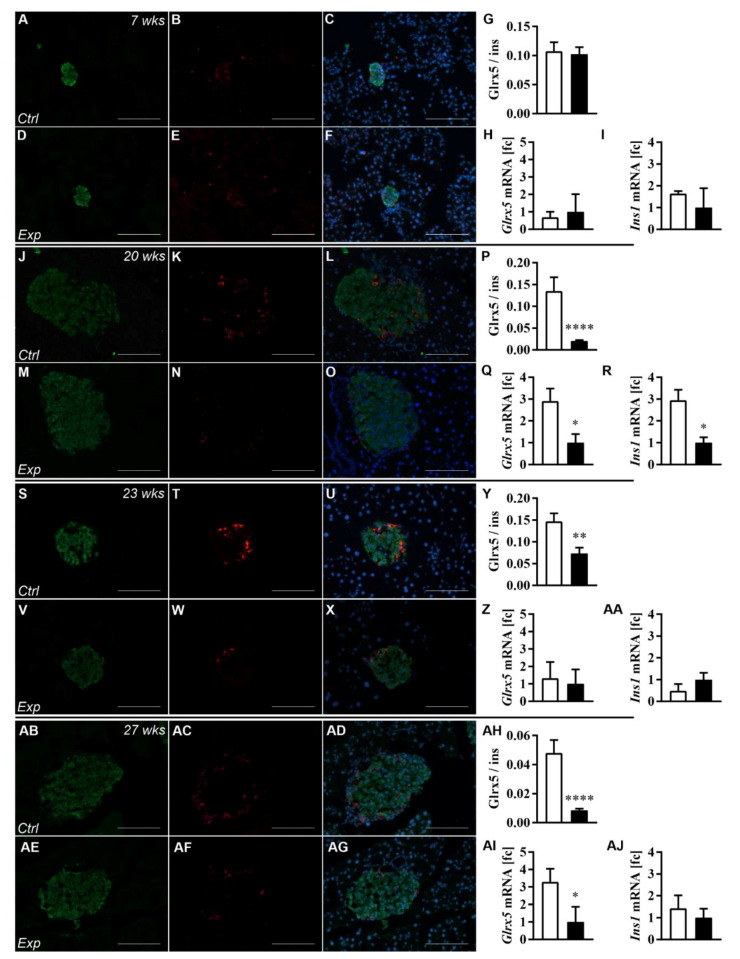
Immunohistological and Ins1 and Glrx5 mRNA expression analysis. The immunohistological staining patterns of insulin and Glrx5 were analyzed at 7, 20, 23, and 27 weeks of age. Glrx5 staining was quantified by calculating the ratio of the Glrx5/insulin staining area. The mRNA expression of *Ins1* and *Glrx5* was studied using qRT-PCR. (**A**–**F**, **J**–**O**, **S**–**X**, **AB**–**AG**) Representative images taken from the immunohistological analysis of insulin and Glrx5 presenting size-matched representative islets of both groups of mice ((**A**–**C**) controls, 7 weeks; (**D**–**F**) HFD, 7 weeks; (**J**–**L**) controls, 20 weeks; (**M**–**O**) HFD, 20 weeks; (**S**–**U**) controls, 23 weeks; (**V**–**X**) HFD, 23 weeks; (**AB**–**AD**) controls, 27 weeks; (**AE**–**AG**) HFD, 27 weeks). Green = insulin, red = Glrx5, blue = nuclei. Scale bars represent 100 µm. Images were taken at 200× magnification. *n* = 33–67 islets of three to four mice/timepoint/group. (**G**,**P**,**Y**,**AH**) The quantification of Glrx5 staining. (**H**,**Q**,**Z**,**AI**) The islet mRNA expression of Glrx5 and (**I**,**R**,**AA**,**AJ**) of Ins1 given as fold change controls vs. HFD for each time point. *n* = six mice/timepoint/group. For each graph, the white bars represent the controls, and the black bars represent the experimental group. **** denotes *p* < 0.0001, ** *p* < 0.005, and * *p* < 0.05 (two-way ANOVA).

Having ensured a diabese phenotype, we aimed to study the effects of dietary changes on islet Glrx5 content. Immunohistology was employed on harvested pancreases and mRNA expression was assessed in isolated islets. The optical assessment of the islets of Langerhans was conducted. No obvious difference in islet morphology and size could be detected when age-matched HFD and control animals were compared (Figure 3A–C controls vs. D–F HFD at 7 weeks, J–L controls vs. M–O HFD at 20 weeks, S–U controls vs. V–X HFD at 23 weeks, AB–AD controls vs. AE–AG HFD at 27 weeks). The quantification of the mean islet area by immunohistology confirmed this observation, revealing no significant difference between both groups of mice (data not shown). The Glrx5 staining pattern indicated a loss of Glrx5 staining in terms of density and intensity in islets of HFD mice throughout the study period. The quantification showed a significantly reduced Glrx5-to-insulin ratio in HFD animals from 20 weeks of age onwards when compared with controls. Interestingly, after switching the diet from the HFD to CD from 20 to 23 weeks of age, HFD mice’s islets presented a recovery of Glrx5 content followed by a pronounced decrease towards the end of the study after 4 more weeks of HFD feeding (Glrx5-to-insulin ratio: 0.107 ± 0.103 vs. 0.102 ± 0.076, n.s. at 7 weeks; 0.135 ± 0.212 vs. 0.02 ± 0.019, **** *p* < 0.0001 at 20 weeks; 0.147 ± 0.104 vs. 0.056 ± 0.081, ** *p* < 0.005 at 23 weeks; 0.048 ± 0.058 vs. 0.008 ± 0.007, **** *p* < 0.0001 at 27 weeks, Figure 3G,P,Y,AH). *Glrx5* mRNA expression was found to follow a similar pattern. The HFD group exhibited a significantly lower expression after both stages of HFD feeding at 20 and 27 weeks of age. The switch to CD resulted in a recovery of expression as measured at 23 weeks of age (fold change control vs. HFD group 0.69-fold, n.s., at 7 weeks, 2.91-fold, * *p* < 0.05, at 20 weeks, 1.32-fold, n.s., at 23 weeks, and 3.29-fold, * *p* < 0.05, at 27 weeks, Figure 3H,Q,Z,AI). *Ins1* mRNA expression correlated with this pattern and the diabese phenotype (fold change control vs. HFD group 1.64-fold, n.s., at 7 weeks, 2.95-fold, * *p* < 0.05, at 20 weeks, 0.49-fold, n.s., at 23 weeks, and 1.43-fold, * *p* < 0.05, at 27 weeks, Figure 3I,R,AA,AJ).

### 3.3. Elevated ROS Production and Impaired GSIS in HFD-Fed Mice

**Figure 4:**
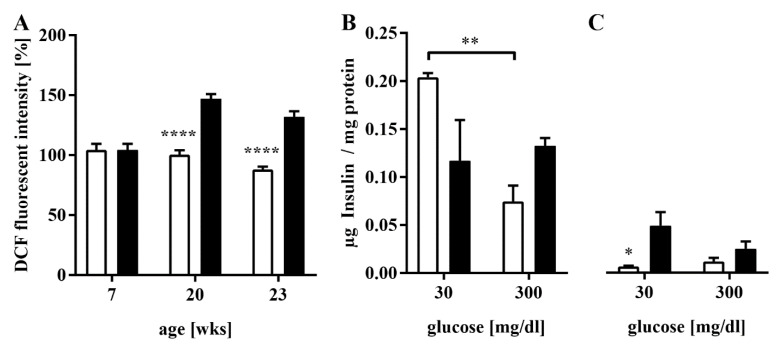
Pancreatic islet ROS production and glucose stimulation. ROS were quantified in pancreatic islets from animals of both groups at 7, 20, and 23 weeks of age by DCFH-DA staining and analysis with a confocal microscope. The glucose stimulation and consecutive measurements of insulin in islet lysates and the medium were conducted in both groups at 27 weeks of age. (**A**) The ROS production of primary islets at 7, 20, and 23 weeks of age. *n* = 63–65 islets of three mice/timepoint/group. (**B**,**C**) The amount of insulin in the (**B**) lysates and (**C**) culture medium of the islets before and after stimulation with 300 mg/dL glucose. *n* = five islets of three mice/timepoint/group, four runs. For each graph, the white bars represent the controls, and the black bars represent the experimental group. **** denotes *p* < 0.0001, ** *p* < 0.005, and * *p* < 0.05 (two-way ANOVA).

ROS production was significantly increased in the islets of the HFD group at 20 and 23 weeks of age (99.8 ± 24.5 vs. 148.5 ± 29.5%, **** *p* < 0.0001 at 20 weeks; 88 ± 15 vs. 131.7 ± 24.2%, **** *p* < 0.0001 at 23 weeks, Figure 4A). We decided to conduct a glucose stimulation and protein analysis instead of ROS measurements at 27 weeks of age. The amount of insulin in the islets of HFD mice showed a non-significant increase when stimulated with glucose, whereas control islets featured a significant drop in cellular insulin (0.07 ± 0.02 vs. 0.2 ± 0.01 µg/mg, ** *p* < 0.005, Figure 4B). The level of insulin in the culture medium increased after stimulating the control islets, whereas stimulating the experimental group’s islets even showed a decrease (0.07 ± 0.004 vs. 0.05 ± 0.05 µg/mg, * *p* < 0.05, Figure 4C). Results indicate elevated basal insulin secretion and confirm defective GSIS, as detected in IPGTT.

### 3.4. FFA Mediate the Loss of Glrx5 in MIN6 Cells

**Figure 5:**
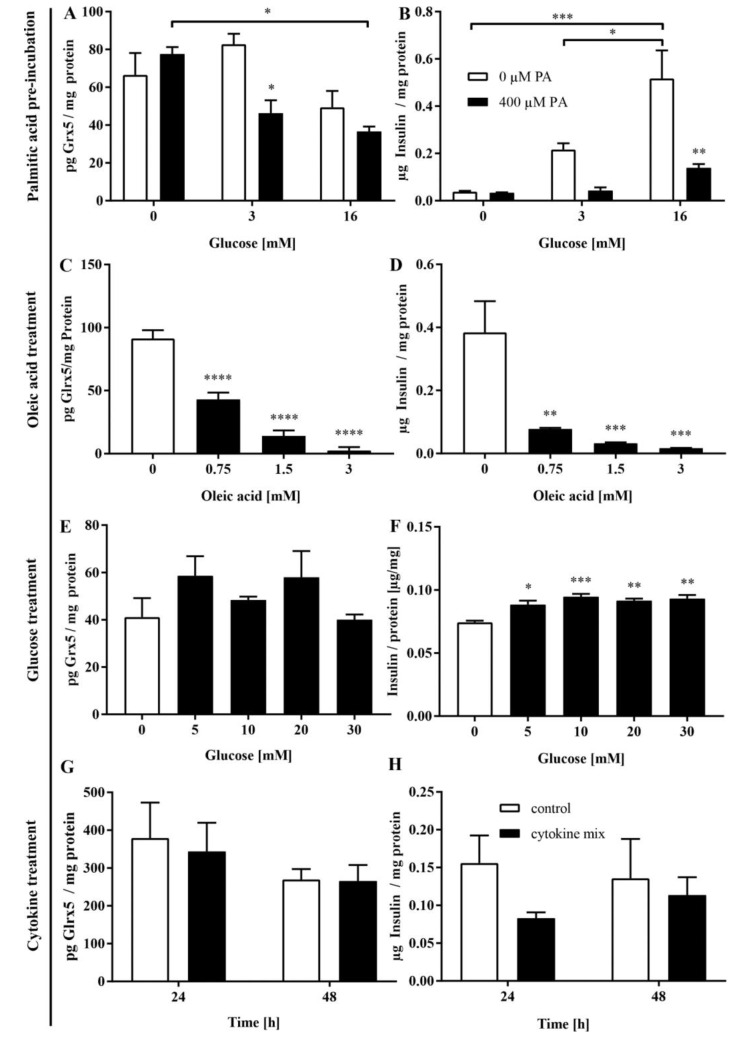
MIN6 Glrx5 level and insulin secretion. (**A**,**B**) Diabetic conditions were modelled in MIN6 cells employing a 48 h period of pre-incubation with 400 µM palmitic acid (PA) followed by 1 h of starvation and exposure to increasing concentrations of glucose for 24 h. (**C**,**D**) Furthermore, we employed a treatment with 0.75, 1.5, or 3 mM oleic acid for 24 h, (**E**,**F**) and a 2 h period of starvation followed by exposure to 5, 10, 20, or 30 mM glucose for 24 h, or (**G**,**H**) a cytokine mix (10 ng/mL TNF-α, 5 ng/mL IL-1β, and 100 ng/mL IFN-γ) for 24 or 48 h, respectively. The protein level of Glrx5 in lysate and insulin in the culture medium of β-cells exposed to (**A**,**B**) glucose for 24 h after and without pre-incubation with 400 µM palmitate and 1h of starvation; (**C**,**D**) oleic acid for 24 h; (**E**,**F**) glucose for 24 h after 2 h of starvation; and (**G**,**H**) a mix of 10 ng/mL TNF-α, 5 ng/mL IL-1β, and 100 ng/mL IFN-γ for 24 or 48 h. Insulin and Glrx5 were measured by ELISA and normalized by total protein. The white bars represent the controls, and the black bars represent the (pre)-treated cells. *n* = 3–5. **** denotes *p* < 0.0001, *** *p* < 0.001, ** *p* < 0.005, and * *p* <0.05 (one-/two-way ANOVA).

To model the observed fluctuation of blood FFA levels in vitro, we pre-incubated MIN6 cells with 400 µM palmitate for 48 h followed by 1 h of starvation and consecutive exposure to glucose (3 and 16 mM) for 24 h. Although the Glrx5 protein level was not significantly affected by exposure to glucose alone, pre-treated cell glucose dependently featured a significantly mitigated level of Glrx5 (82.8 ± 9.5 vs. 46.3 ± 11.8 pg/mg, * *p* < 0.05 at 3 mM glucose, Figure 5A). The amount of insulin in the medium was significantly lower in pre-treated cells, indicating a correlation between insulin secretion and Glrx5 (0.56 ± 0.14 vs. 0.14 ± 0.03 µg/mg, ** *p* < 0.005 at 16 mM glucose, Figure 5B). The exposure to oleic acid alone led to similar results. In a dose-dependent manner, the amount of Glrx5 (91.32 ± 13.11 vs. 43.03 ± 10.91 vs. 20.82 ± 7.56 vs. 8.76 ± 0.85 pg/mg, **** *p* < 0.0001, Figure 5C) as well as the amount of insulin in the medium (0.38 ± 0.2 vs. 0.08 ± 0.01 vs. 0.03 ± 0.01 vs. 0.02 ± 0.002 µg/mg, ** *p* < 0.005 at 0.75 mM, *** *p* < 0.001 at 1.5 and 3 mM, Figure 5D) decreased. Similar results were observed for palmitic acid (data not shown). To separate the effects of FFA and hyperglycemia on Glrx5 in β-cells, incubation with glucose in varying concentrations for 24 h was conducted after 2 h of starvation. No significant impact on Glrx5 was detected (41.17 ± 13.93 vs. 58.63 ± 14.24 vs. 48.44 ± 2.43 vs. 58.05 ± 19.05 vs. 40.11 ± 3.85 pg/mg, n.s., Figure 5E), whereas insulin in the culture medium increased (0.074 ± 0.002 vs. 0.089 ± 0.001 vs. 0.094 ± 0.004 vs. 0.091 ± 0.003 vs. 0.093 ± 0.005 µg/mg, * *p* < 0.05 at 5 mM glucose, *** *p* < 0.001 at 10 mM glucose, ** *p* < 0.005 at 20 and 30 mM glucose, Figure 5F). Since inflammation is an important factor in the diabetic metabolism, we also studied the effect of inflammatory cytokines. Cells were exposed to a cytokine mix consisting of 10 ng/mL TNF-α, 5 ng/mL IL-1β, and 100 ng/mL IFN-γ [30] for 24 and 48 h, respectively. As displayed in Figure 5G, no significant change in Glrx5 was observed upon treatment (385.1 ± 190.6 vs. 352 ± 154.9 pg/mg after 24 h, 275.1 ± 57.64 vs. 268.4 ± 84.34 pg/mg after 48 h, n.s.). The insulin in the culture medium declined non-significantly (0.16 ± 0.07 vs. 0.08 ± 0.02 µg/mg at 24 h; 0.14 ± 0.1 vs. 0.11 ± 0.05 µg/mg at 48 h, n.s., Figure 5H).

**Figure 6:**
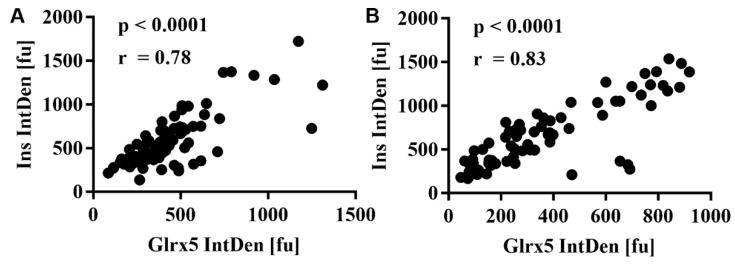
Cytological analysis of MIN6 insulin and Glrx5 content. MIN6 cells exposed to 1.5 mM oleic acid for 24 h were stained for insulin and Glrx5 and compared with the controls. Staining was quantified by measuring the integrated density using ImageJ software. The values for insulin and Glrx5 were correlated. (**A**) The correlation of insulin with Glrx5 in the control cells and (**B**) after treatment with 1.5 mM oleic acid for 24 h. *n* = 4 (9–35 cells/run, Pearson).

The correlation between MIN6 insulin and Glrx5 content on a single-cell level was studied by employing immunocytochemistry. The quantification of insulin staining correlated well with Glrx5 staining both without treatment (Figure 6A, r = 0.78, **** *p* < 0.0001) and after 24 h of exposure to 1.5 mM oleic acid (Figure 6B, r = 0.83, **** *p* < 0.0001).

### 3.5. The Loss of Glrx5 Is Correlated with Mitigated ATP and Impaired O_2_ Flux in the Respiratory Chain

**Figure 7:**
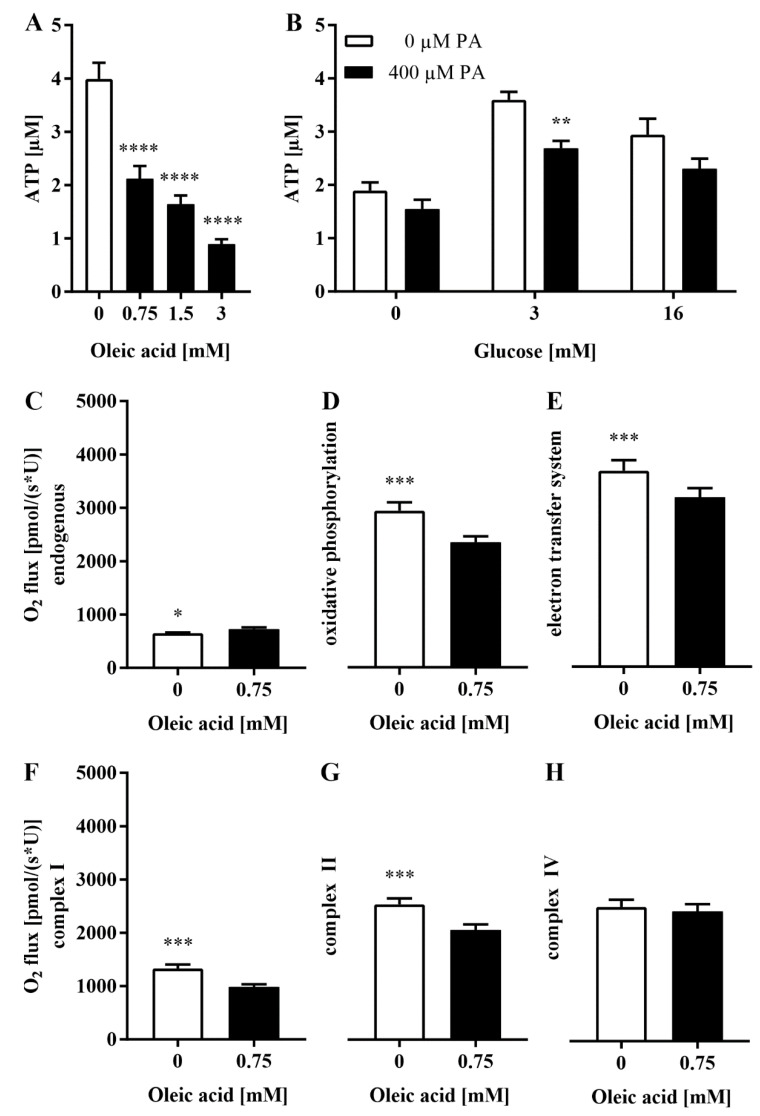
ATP production and the O_2_ flux in the respiratory chain after FFA treatment. Cellular ATP levels were analyzed employing a luciferase-based bioluminescence assay. Respiratory states were measured with an Oxygraph and an O_2_ probe. (**A**) ATP after exposure to oleic acid for 24 h or (**B**) after pre-incubation with 400 µM palmitate, 1 h of starvation, and exposure to glucose. (**C**–**H**) The O_2_ flux in the respiratory chain after exposure to 0.75 mM oleic acid for 24 h. The white bars represent the controls, and the black bars represent the (pre-)treated cells. *n* = 12. **** denotes *p* < 0.0001, *** *p* < 0.001, ** *p* < 0.005 and * *p* < 0.05 (one-way ANOVA/unpaired *t*-test).

The impact of diminished levels of Glrx5 on the respiratory chain was analyzed by the available amount of ATP in MIN6 cells. A dose-dependent exposure to oleic acid as well as pre-incubation with 400 µM palmitate led to a significant decrease in ATP (3.99 ± 0.87 vs. 2.12 ± 0.63 vs. 1.6 ± 0.45 vs. 0.89 ± 0.25 µM, **** *p* < 0.0001, Figure 7A and 3.6 ± 0.43 vs. 2.69 ± 0.46 µM at 3 mM glucose, ** *p* < 0.005, Figure 7B). To gain insight into the reason behind this energy deficiency, we examined the O_2_ flux through the respiratory chain. Endogenous respiration was significantly increased in treated cells, indicating an increased substrate turnover which was less efficient due to increased uncoupling already in the basal state (650.5 ± 41.86 vs. 727 ± 109 pmol/(s*U), * *p* < 0.05, Figure 7C). The maximum possible O_2_ flux was measured upon saturation with respective substrates and revealed a significantly decreased maximum capacity of oxidative phosphorylation in treated cells (2935 ± 537.2 vs. 2340 ± 399.4 pmol/(s*U), *** *p* < 0.001, Figure 7D). Maximum electron transfer capacity, analyzed after adding an uncoupler, was also significantly lower after exposure to oleic acid (3672 ± 784 vs. 3173 ± 603.1 pmol/(s*U), *** *p* < 0.001, Figure 7E). Ultimately, the O_2_ flux through both complex I and II was significantly diminished (1332 ± 230 vs. 984.5 ± 156.7 pmol/(s*U), *** *p* < 0.001, Figure 7F, 2537 ± 394.7 vs. 2057 ± 370 pmol/(s*U), *** *p* < 0.001, Figure 7G), whereas complex IV was not affected (2516 ± 416.8 vs. 2437 ± 375 pmol/(s*U), n.s., Figure 7H).

## 4. Discussion

It is acknowledged that chronic hyperglycemia and elevated FFA exert deleterious effects on the pancreatic β-cell, and continuous advances in research have identified numerous underlying pathways and mechanisms [31,32]. In particular, mitochondria, which are essential for insulin secretion due to their crucial role in fuel metabolism, are affected significantly, presenting a severely altered expression of carrier proteins [33], morphology [34], and increased volume [35]. Little is known about the role of mitochondrial glutaredoxins. We have previously reported a loss of Glrx5 in islets of monogenetically diabese db/db-mice [21,25]. However, this model is hardly comparable with type 2 diabetes mellitus, as it occurs in human subjects, since this complex metabolic disorder is of a polygenetic and multifactorial origin. In this study, we could demonstrate that the loss of Glrx5 is also apparent in an HFD-induced murine model of diabesity. Moreover, standard chow led to the reconstitution of islet Glrx5 and ameliorated glucose tolerance (Figure 2 and Figure 3). Serum FFA levels correlated with the diabese phenotype and the amount of islet Glrx5. This leads to the conclusion that FFA mediate the loss of Glrx5 in vivo. This hypothesis was confirmed in vitro, where the exposure of MIN6 cells to FFA led to a loss of Glrx5 concomitant with a decrease in insulin secretion, while treatment with glucose or inflammatory cytokines had no such effect (Figure 5 and Figure 6).

It is generally recognized that FFA can exert both positive and negative effects on the β-cell’s viability and insulin secretion, and are dependent on their carbon chain length and concentration. Furthermore, exposure time and the presence of hyperglycemia or inflammatory cytokines modulate their effects. An acute increase in FFA is linked to increased β-cell mass and insulin secretion mediated through the FFA receptor GPR40/FFAR [36,37] and PPAR [38]. The chronic elevation of FFA fuels insulin resistance and exerts detrimental effects on insulin secretion [39], gene expression [40], and β-cell survival [41] as well, mostly due to increased oxidative and endoplasmic reticulum stress, GLUT translocation, and inflammation [7,42]. However, the impact of FFA on basal insulin release and GSIS is differential. GSIS is inhibited but basal insulin is even increased [39,43,44] due to the decreased activity of citrate synthase which ultimately results in increased hexokinase activity, enhancing β-cell glucose usage [45]. Hyperglycemia enhances the detrimental effects of FFA since the respiratory chain uncoupling by UCP2 requires high membrane potential as reached at high glucose concentrations [46]. Excessive uncoupling favors mitochondrial dysfunction. Elevated glucose turnover inhibits AMPK [47] and increases the concentration of malonyl-CoA. An abundance of malonyl-CoA diminishes the oxidation of FFA by inhibiting the mitochondrial fatty acid transporter CPT1 [48]. The subsequent accumulation of FFA activates caspases [49], promoting apoptosis. Consistently, our findings show both enhanced ROS production and elevated basal insulin secretion with the failure of GSIS in pancreatic islets (Figure 4) after the HFD-induced abundance of FFA. In human subjects with normal glucose tolerance, elevated fasting FFA levels were associated with decreased insulin secretion and the risk of developing impaired glucose tolerance [50].

Our data indicate a significant loss of ATP and a decrease in oxidative phosphorylation and O_2_ flux through the respiratory chain (Figure 7) as correlates for mitochondrial dysfunction. Similar observations were made in islets of C57BL/6J after 12 weeks of high-fat diet feeding. Islets featured diminished glucose oxidation upon stimulation with glucose and increased palmitate oxidation in the basal state, indicating a switch in substrates during this insulin-resistant state [35]. Comparable findings were achieved in human islets [34]. The availability of ATP is a prerequisite for insulin secretion, but GSIS requires mitochondrial respiration [51,52]. The main source of ATP production depends on glucose responsiveness in MIN6 cells. Less responsive MIN6 cells allocate ATP mainly through nonoxidative pathways [53], whereas insulin secretion is dependent on oxidative phosphorylation [54,55]. This was demonstrated for MIN6 cells which become less sensible to glucose and have diminished ATP content as well as low lipid oxidation at high passages [56]. In vivo studies even identified heterogenic islet populations marked by varying glucose sensitivity and mitochondrial respiration in mice and humans [57].

Most interestingly, the O_2_ flux was significantly impaired in both complex I and II, which both rely on Fe/S clusters, but not in complex IV, which does not contain any Fe/S clusters (Figure 7). Glrx5 is known to be essential for the activity of Fe/S enzymes [58,59]. Camaschella et al. described diminished cytosolic and mitochondrial aconitase activity and expression in a Glrx5-deficient human subject [16] which was also found in murine osteoblasts [12]. Corbett et al. had previously related the loss of mitochondrial aconitase activity with cytokine-induced NO production and abolished insulin secretion in rat β-cell and human islets [60,61,62]. Diminished Fe/S cluster biosynthesis was shown to mitigate the function of Cdkal1, an Fe/S cluster enzyme crucial for proinsulin processing in iron-regulatory protein 2-deficient mice and rat insulinoma cells [63]. These data indicate that the impact of iron metabolism on insulin production and secretion is more complex and not limited to the abundance of free iron, which is well known to act in a diabetogenic manner. Rather, this suggests that the impaired Fe/S clusters might function as a possible link between mitigated mitochondrial function and the deprivation of Glrx5.

There are limitations to our study. Although our data indicate a 90% reduction in Glrx5 after exposure to oleic acid in MIN6 cells, the absolute amount of cellular Glrx5 was very low and we did not study iron metabolism in our models; thus, the question of whether Glrx5 is a cause of mitochondrial dysfunction and impaired insulin secretion remains open. General toxic effects of diabetic stressors on the mitochondria might account for our observations. Thus, further studies, especially employing models with Glrx5 knockout and overexpression and investigations focusing on human material, need to be conducted to further characterize the significance of Glrx5 for the pancreatic β-cell.

## 5. Conclusions

We deliver further evidence for the role of Glrx5 in β-cell deficiency in type 2 diabetes mellitus. Our data indicate that Glrx5 is downregulated by FFA and that recovery can be achieved by diet. We propose mitochondrial dysfunction as a possible underlying mechanism linking the loss of Glrx5 to mitigated insulin secretion.

## Data Availability

All data are included within this manuscript.
